# Postdiagnostic physical activity, sleep duration, and TV watching and all-cause mortality among long-term colorectal cancer survivors: a prospective cohort study

**DOI:** 10.1186/s12885-017-3697-3

**Published:** 2017-10-25

**Authors:** Ilka Ratjen, Clemens Schafmayer, Romina di Giuseppe, Sabina Waniek, Sandra Plachta-Danielzik, Manja Koch, Greta Burmeister, Ute Nöthlings, Jochen Hampe, Sabrina Schlesinger, Wolfgang Lieb

**Affiliations:** 1 Institute of Epidemiology, Christian-Albrechts-University of Kiel, University Hospital Schleswig-Holstein, Niemannsweg 11 (Haus 1), 24105 Kiel, Germany; 2 Department of General and Thoracic Surgery, University Hospital Schleswig-Holstein, Kiel, Germany; 3 Department of Nutrition, Harvard T.H. Chan School of Public Health, Boston, MA USA; 4 Nutritional Epidemiology, Department of Nutrition and Food Science, Rheinische Friedrich-Wilhelms-University Bonn, Bonn, Germany; 5 Medical Department 1, University Hospital Dresden, Technical University Dresden, Dresden, Germany; 6 Institute of Biometrics and Epidemiology, German Diabetes Center at Heinrich Heine University, Leibniz Institute for Diabetes Research, Düsseldorf, Germany

**Keywords:** Postdiagnostic, Physical activity, Sleep duration, TV watching, Colorectal cancer, Survivors, Mortality

## Abstract

**Background:**

Lifestyle recommendations for cancer survivors are warranted to improve survival. In this study, we aimed to examine the association of total physical activity, different types of physical activity, hours of sleeping at day and night, and hours spent watching television (TV) with all-cause mortality in long-term colorectal cancer (CRC) survivors.

**Methods:**

We assessed physical activity in 1376 CRC survivors (44% women; median age, 69 years) at median 6 years after CRC diagnosis using a validated questionnaire. Multivariable-adjusted Cox regression models were used to estimate hazard ratios (HRs) for all-cause mortality according to categories of physical activities, sleep duration, and TV watching.

**Results:**

During a median follow-up time of 7 years, 200 participants had died. Higher total physical activity was significantly associated with lower all-cause mortality (HR: 0.53; 95% CI: 0.36–0.80, 4th vs. 1st quartile). Specifically, sports, walking, and gardening showed a significant inverse association with all-cause mortality (HR: 0.34; 95% CI: 0.20–0.59, HR: 0.65; 95% CI: 0.43–1.00, and HR: 0.62; 95% CI: 0.42–0.91, respectively for highest versus lowest category). Individuals with ≥2 h of sleep during the day had a significantly increased risk of all-cause mortality compared to individuals with no sleep at day (HR: 2.22; 95% CI: 1.43–3.44). TV viewing of ≥4 h per day displayed a significant 45% (95% CI: 1.02–2.06) higher risk of dying compared to ≤2 h per day of watching TV.

**Conclusions:**

Physical activity was inversely related to all-cause mortality; specific activity types might be primarily responsible for this association. More hours of sleep during the day and a higher amount of TV viewing were each associated with higher all-cause mortality. Based on available evidence, it is reasonable to recommend CRC survivors to engage in regular physical activity.

**Electronic supplementary material:**

The online version of this article (10.1186/s12885-017-3697-3) contains supplementary material, which is available to authorized users.

## Background

In 2012, there were nearly 1.4 million people diagnosed with colorectal cancer (CRC) and it is predicted that by 2035 the number of cases will increase to 1.36 million for men and 1.08 million for women worldwide [1]. On a parallel note, death rates of CRC have fallen by on average 2.5% each year from 2005 to 2014 in the US and the 5-year relative survival is about 64.9% in the US and about 63% in Germany [2, 3]. Rising survival rates and increasing numbers of newly diagnosed cases lead to a growing group of CRC survivors [4]. Therefore, as outlined by the World Cancer Research Fund [5], there is rising interest in to what extent behavioral factors affect the course of the disease and survival of patients with CRC [6].

Regular physical activity has a broad range of beneficial health effects, e.g., on obesity and other cardiovascular risk factors [7] and is associated with better overall survival in the general population and in many patient groups [8, 9]. Additionally, physically active people have a lower risk of developing different forms of cancer [10], including colon cancer [11]. A meta-analysis of 52 studies reported a risk reduction of colon cancer incidence of about 24% in physically active men and of about 21% in active women compared to inactive people [11]. Besides, evidence is growing that physical activity is also safe and well-tolerated by cancer patients during and after treatment [12, 13]. Furthermore, exercise has been shown to increase quality of life and to improve physical functioning among cancer survivors [14, 15].

Prior studies have investigated the association between physical activity and mortality in CRC patients and reported 25–63% lower disease-specific and all-cause mortality for more active as compared to less active patients after CRC diagnosis [16–23]. However, previous studies focused on physical activity that was assessed relatively shortly after diagnosis (range: 5 months to 4.2 years median) [16–23] and less is known about the impact of different types of physical activity on mortality of CRC survivors. Two studies examined the relation of postdiagnostic television (TV) viewing with all-cause mortality in CRC survivors and found a 25–45% increase in mortality for the highest category of TV watching, but statistical significance was not reached [16, 24].

Cancer survivors, especially CRC survivors, are mostly elderly. Colon and rectum cancer are most frequently diagnosed among persons aged 65–74 years [3]. In this predominant age group, physical activity can imply a lot of advantages in health, quality of life, and social life but might also represent a practical challenge for some people due to age-related comorbidities [25]. Therefore, resulting health benefits of physical activity should be investigated thoroughly.

In this study, we assessed the association of postdiagnostic total physical activity, different types of physical activity (‘sports’, ‘cycling’, ‘walking’, ‘gardening’, ‘housework, home repair, and stair climbing’), hours of sleeping at night and day, and time spent watching TV with all-cause mortality among CRC long-term survivors.

## Methods

### Study sample

Between 2004 and 2007, a total of 2733 patients with histologically confirmed CRC (diagnosed between 1993 and 2005) were recruited by the biobank PopGen after identification through medical records from surgical departments in 23 hospitals in Northern Germany and through the regional cancer registry. Detailed information on this sample has been reported previously [14, 26, 27]. Patients filled in a questionnaire about clinical characteristics and socio-demographic and selected lifestyle factors. The study protocol was approved by the institutional ethics committee of the Medical Faculty of Kiel University and written informed consent was obtained from all study participants.

Between 2009 and 2011, 2263 patients who initially agreed to be re-contacted were asked to complete another questionnaire about clinical and socio-demographic factors, a food frequency questionnaire (FFQ) [28] with additional questions about physical activity [29], and a questionnaire on health-related quality of life (HrQol) [30]. Of the 2263 participants contacted, 1452 (64%) responded to the FFQ and to the questions on physical activity. Compared to non-responders (*n* = 694, 25.4%) and deceased (*n* = 354, 13.0%) individuals of the initial study sample of 2733 individuals, the participants who completed the physical activity questionnaire were younger at baseline and at CRC diagnosis, reported more often a family history of CRC, and had less often metastases or other types of cancer [14]. We excluded individuals with missing information on year of diagnosis (*n* = 21) and vital status (*n* = 21), those with implausible length of follow-up (*n* = 3), and participants with a diagnosis of small intestine cancer instead of CRC (*n* = 3). Finally, to eliminate outliers (extreme values) of physical activity, we excluded individuals above the 98th percentile of total physical activity (*n* = 28), leaving an analytical sample of 1376 participants (61% of the initial study sample contacted for follow-up).

### Physical activity assessment

A validated questionnaire was applied to assess physical activity during the past 12 months [29]. From these questions, average hours per week spent with different activities, including walking, cycling, sports (physical exercise except for cycling), and gardening, each separately for summer and winter, as well as housework (e.g. cooking, washing, cleaning), and home repair (do-it-yourself) were enquired. Additionally, stair climbing defined as floors per day, hours of sleeping at night and day, respectively and hours per day spent watching TV were quantified. Metabolic equivalent of task (MET) values, according to the 2000 Compendium of Physical Activity [31], were assigned to each corresponding activity [32]. One MET is defined as the energy expenditure for sitting quietly and MET-values are the ratio of the metabolic rate for a specific activity divided by the resting metabolic rate [31]. Thus, the number of hours per week spent with each activity (where applicable, the mean number of hours was calculated from summer and winter activities) were multiplied by the respective MET-values (walking: 3.0, cycling: 6.0, sports: 6.0, gardening: 4.0, housework: 3.0, home repair: 4.5, stair climbing: 8.0) [31, 32]. To derive MET-hours per week of total physical activity, the MET-hours of walking, cycling, sports, gardening, housework, home repair, and stair climbing were summed up.

### Clinical and socio-demographic characteristics

The self-administered questionnaires about clinical characteristics included questions related to tumor location (colon, rectum, both lesions), occurrence of metastases or other types of cancer (both reported at baseline and physical activity assessment), and neoadjuvant and adjuvant cancer therapies. We validated these self-reported clinical data (tumor location, type of therapy, metastases) against medical records in a subset of 181 participants and observed overall good agreement (87% concordance). Among socio-demographic factors, sex, age at diagnosis, age at physical activity assessment, smoking status (never, former, current) at physical activity assessment, and postdiagnostic body weight and height at baseline and physical activity assessment were self-reported. Body Mass Index (BMI; kg/m^2^) was defined as weight divided by the square of height in meters. Total energy intake has been calculated from FFQ data [28] and global health-related quality of life (gHrQol; score ranging from 0 to 100) was assessed by the EORTC-QLQ C30 (version 3.0) [30].

### Vital status ascertainment

Vital status ascertainment has been described in detail elsewhere [27]. In 2016, vital status of all participants was updated via population registries and date of death was recorded if participants were deceased (date of death could be verified for all cases). The date of physical activity assessment was used as starting point for follow-up of this study and follow-up ended with date of death or last vital status assessment whichever came first.

### Statistical analyses

Participant characteristics were compared across quartiles of total physical activity. Differences in categorical variables were tested using a chi-squared test and differences in distributions of continuous variables were tested with the Wilcoxon ranksum test.

The Kaplan-Meier curves and log-rank test were used to investigate (unadjusted) differences in the survival time distribution of CRC survivors according to quartiles of total physical activity.

HRs and 95% CIs for the association of total physical activity, different types of physical activity, hours of sleeping at night or day, and hours per day of watching TV with all-cause mortality were estimated using Cox proportional hazards regression models with age as the underlying time variable. Total physical activity was modeled in quartiles and individual activities, sleep duration, and TV watching were modeled in appropriate categories of MET-hours/week or hours/day. For sports, cycling, and gardening, categories of 0, >0–10, >10–20, and >20 MET-hours/week were chosen similar to a recent analysis in a German study that used the same physical activity questionnaire [33]. For walking and activities from housework, home repair, and stair climbing, categories of 0–10, >10–20, >20–30, and >30 MET-hours/week were used because these activities were reported with an overall higher amount of MET-hours/week and a low prevalence of 0 MET-hours/week. The categories for hours of sleeping at night (≤6, 7–8, and ≥9 h/day) were chosen based on sleep time duration recommendations of the National Sleep Foundation [34]. Categories for hours of sleeping at day (0, >0- < 1, 1- < 2, and ≥2 h/day) and hours of watching TV (≤2, >2- < 4, and ≥4 h/day) were chosen based on the distribution of reported values. HRs were calculated for each quartile/category using the first quartile/lowest category as the referent, except for sleeping at night where the recommended optimal level of 7–8 h/day was used as the referent. To control for confounding, all models were adjusted for sex and age at physical activity assessment. A second model was additionally adjusted for BMI at physical activity assessment (continuous in kg/m^2^), survival time from CRC diagnosis until physical activity assessment (continuous in years), smoking status (never, former, current, unknown), alcohol intake (continuous in g/day), tumor location (colon, rectum, both, unknown), occurrence of metastases (yes, no, unknown), occurrence of other cancers (yes, no, unknown), and chemotherapy (yes, no, unknown). We also considered the presence of a stoma and family history of CRC as potential confounders but decided not to include those in the final model because the results did not change substantially (<10%). In addition, the individual activities ‘cycling’, ‘sports’, ‘walking’, ‘gardening’, and ‘housework, home repair, and stair climbing’ were mutually adjusted for. Furthermore, hours of sleeping at night and hours of sleeping at day were mutually adjusted for. Time spent watching TV was additionally adjusted for total physical activity. We tested the proportional hazards assumption by the Schoenfeld residuals method and by including time-dependent variables in the models. Because age, BMI, and metastases did not meet the proportional hazards assumption, respective time-dependent multiplicative interaction terms (time x age, time x BMI, time x metastases) were included in the models. Tests for linear trend across quartiles or categories were performed by modeling the median value for each quartile/category as a continuous variable and by including this variable in the respective Cox regression model.

The degree of nonlinearity in the association of total physical activity with all-cause mortality was evaluated with restricted cubic spline regression, fitted with four knots (5th, 35th, 65th, and 95th percentile [35]) and a reference point located at the median (44 MET-hours/week) of the reference group (Quartile 1) of the main analysis. This model was adjusted for the same covariates as the main model (described above).

In subgroup analyses, HRs and 95% CIs of all-cause mortality for the fourth versus the first quartile of total physical activity were calculated stratified by sex (men vs. women), median age at physical activity assessment (<69 vs. ≥69 years), BMI (<25 vs. 25 - <30 vs. ≥30 kg/m^2^), tumor location (colon vs. rectum), occurrence of metastases (yes vs. no), and smoking status (never vs. ever). We additionally stratified by the median of gHrQol (<75 vs. ≥75) to assess potential differences in the association of physical activity with all-cause mortality between individuals with a higher and a lower gHrQol. Respective multiplicative interaction terms were tested in the multivariable-adjusted models by including the cross product of total physical activity and the potential effect modifier.

To investigate the robustness of our findings, sensitivity analyses were performed. To account for reverse causality, we examined the association of postdiagnostic total physical activity with all-cause mortality after excluding CRC survivors who died within 12 months after physical activity assessment. In a second sensitivity analysis, we excluded participants who reported a diagnosis of metastases either at baseline or first follow-up because the occurrence of metastases could influence the ability of being physically active and the survival rate. In another sensitivity analysis we additionally added gHrQol (modeled on a continuous scale) to the multivariable-adjusted model in order to assess the effect of quality of life on the association between physical activity and survival and to further account for potential reverse causality. In addition, it might be possible that complete inactivity could be an indicator for disease status, reflecting individuals with very poor health status. Thus, in a sensitivity analysis, individuals with 0 MET-hours of total physical activity were excluded. In a fifth sensitivity analysis, we additionally adjusted the association of TV watching with all-cause mortality for total energy intake to assess the potential role of high intake of energy-dense foods associated with sedentary time for survival [36].

All statistical analyses were conducted using SAS version 9.4 software (SAS Institute, Inc., NC, USA). Two-sided *p* values of <0.05 were considered statistically significant.

## Results

### Participant characteristics

Characteristics of the overall study population and stratified by quartiles of postdiagnostic total physical activity are provided in Table 1. Of the 1376 individuals, 44% were women, the median age at diagnosis was 62 years, and the median time between CRC diagnosis and physical activity assessment was 6 years. Nearly half of the participants had a tumor located in the colon (48%), 42% had a rectum carcinoma, 17% of the participants reported a diagnosis of metastases, and 21% a diagnosis of other cancers either at baseline or first follow-up. More than half of the study population had only surgery and no other CRC therapy was carried out (Table 1). The study participants reported a median of 100 MET-hours/week (interquartile range: 65–145) of total physical activity. Compared with participants in the first quartile of postdiagnostic total physical activity, participants with a higher amount of total physical activity were more likely to be women, were younger at the time of diagnosis and at physical activity assessment, and had a higher consumption of alcohol (Table 1).

**Table 1:** Characteristics of the overall sample of CRC survivors (*n* = 1376) and according to quartiles of total physical activity (in MET-hours/week)

		Quartiles of total physical activity				
Participant characteristics	Overall sample	Q1 (0–64.5)	Q2 (>64.5–99.7)	Q3 (>99.7–144.9)	Q4 (>144.9)	*p* ^a^
Total no. of individuals, n	1376	344	344	344	344	
No. of deaths, n (%)	200 (15)	85 (25)	47 (14)	33 (10)	35 (10)	<0.0001
Sex, n (%)						
Men	770 (56)	224 (65)	200 (58)	176 (51)	170 (49)	
Women	606 (44)	120 (35)	144 (42)	168 (49)	174 (51)	<0.0001
Age at diagnosis, y	62 (57–66)	63 (57–70)	62 (56–66)	62 (57–66)	61 (56–65)	0.0002
Age at physical activity assessment, y	69 (64–73)	70 (65–77)	69 (64–74)	69 (65–73)	68 (63–72)	0.0006
Time between CRC diagnosis and physical activity assessment, y	6 (5–8)	6 (5–8)	7 (5–8)	7 (5–8)	6 (5–8)	0.37
BMI, kg/m^2^	26.2 (23.8–29.3)	26.6 (24.0–29.4)	26.0 (23.7–29.3)	26.1 (23.8–29.1)	26.0 (23.7–29.2)	0.63
Smoking status, n (%)						
Never	556 (40)	123 (36)	140 (41)	143 (42)	150 (44)	
Former	678 (49)	177 (51)	171 (50)	170 (49)	160 (47)	
Current	121 (9)	37 (11)	30 (9)	26 (8)	28 (8)	
Unknown	21 (2)	7 (2)	3 (1)	5 (1)	6 (2)	0.57
Alcohol intake, g/day	7 (2–20)	5 (1–20)	8 (2–23)	7 (3–18)	7 (2–18)	0.01
Tumor location, n (%)						
Colon	657 (48)	166 (48)	168 (49)	170 (49)	148 (43)	
Rectum	576 (42)	147 (43)	144 (42)	137 (40)	153 (44)	
Both	62 (5)	13 (4)	13 (4)	21 (6)	15 (4)	
Unknown	81 (6)	18 (5)	19 (5)	16 (5)	28 (8)	0.48
Metastases, n (%)						
Yes	234 (17)	70 (20)	48 (14)	54 (16)	56 (16)	
No	908 (66)	207 (60)	248 (72)	227 (66)	226 (66)	
Unknown	234 (17)	67 (19)	48 (14)	63 (18)	62 (18)	0.06
Other Cancer, n (%)						
Yes	292 (21)	73 (21)	79 (23)	68 (20)	72 (21)	
No	1054 (77)	261 (76)	260 (76)	268 (78)	265 (77)	
Unknown	30 (2)	10 (3)	5 (1)	8 (2)	7 (2)	0.84
Therapy, n (%)						
None	721 (52)	182 (53)	191 (56)	168 (49)	180 (52)	
Chemotherapy	305 (22)	85 (25)	68 (20)	80 (23)	72 (21)	
Radiation	45 (3)	6 (2)	18 (5)	11 (3)	10 (3)	
Chemotherapy and radiation	282 (20)	65 (19)	59 (17)	80 (23)	78 (23)	
Unknown	23 (2)	6 (2)	8 (2)	5 (1)	4 (1)	0.18

Values are n (%) or median (interquartile range)

*Abbreviations*: *BMI* body mass index, *CRC* colorectal cancer, *MET* metabolic equivalent of task

^a^Based on chi-squared test for categorical variables and Wilcoxon’s rank-sum test for continuous variables

### Postdiagnostic physical activity, sleep duration, and TV watching and all-cause mortality

After the assessment of physical activity, individuals were followed for a median time period of 7 years. During this period, 200 (14.5%) of the 1376 study participants had died.

**Fig. 1:**
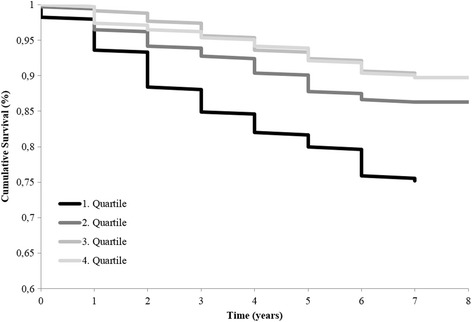
Kaplan-Meier-Curves of overall survival of 1376 CRC survivors according to quartiles of total physical activity. The log-rank *p* value is <0.0001. Abbreviations: CRC, colorectal cancer

**Fig. 2:**
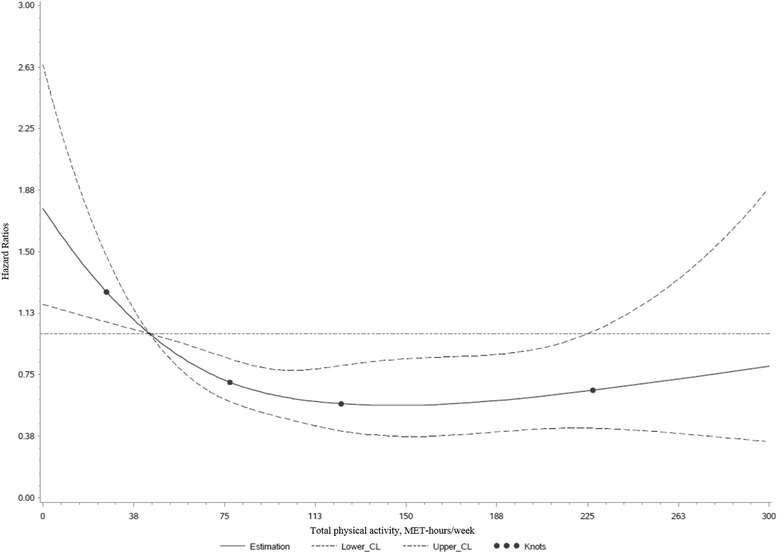
Multivariable-adjusted hazard ratios of all-cause mortality according to total postdiagnostic physical activity in CRC survivors (*n* = 1376), calculated with restricted cubic spline regression. The solid line depicts hazard ratios and the dashed lines are the 95% CIs. The points indicate the knots on 5th, 35th, 65th, and 95th percentiles. The reference value is the median (44 MET-hours/week) of the first quartile of total physical activity. The model was adjusted for sex, age at physical activity assessment, BMI, survival time from CRC diagnosis until physical activity assessment, tumor location, occurrence of metastases, occurrence of other cancer, chemotherapy, smoking status, and alcohol intake. The *p* value for nonlinearity is 0.01 (Wald chi-square test). Abbreviations: BMI, body mass index; CRC, colorectal cancer; MET, metabolic equivalent of task

Figure 1 displays significant differences in the survival time between quartiles of total physical activity (log-rank *p* value <0.0001), in the sense that higher quartiles of activity showed better survival as compared to lower quartiles. However, the difference in survival time between quartiles decreased with increasing quartile displaying less distinct differences between quartiles 3 and 4 with respect to cumulative survival. In a multivariable-adjusted Cox regression model, individuals in quartiles 2 to 4 of total physical activity all displayed statistically significantly longer survival as compared to individuals in the first quartile, with a 47% reduction of all-cause mortality in the fourth quartile (HR: 0.53; 95% CI: 0.36–0.80; p_trend_ = 0.0006; Table 2). Using cubic spline regression, we observed evidence for a statistically significant nonlinear association between total physical activity and all-cause mortality (p_nonlinear_ = 0.01, Wald chi-square test). With increasing physical activity the survival benefit is growing until a plateau is reached around the third quartile (about 130 MET-hours/week; Fig. 2).

**Table 2:** HRs^a^ and 95% CIs of all-cause mortality according to quartiles of total physical activity and according to categories of individual activities, sleep duration, and TV watching in CRC survivors (*n* = 1376)

	Total no. of individuals	No. of deaths	Age- & sex-adjusted HR (95% CI)	Multivariable-adjusted^b^ HR (95% CI)
*MET-hours/week of total physical activity*				
Quartile 1 (0–64.5)	344	85	1.00 (Ref.)	1.00 (Ref.)
Quartile 2 (>64.5–99.7)	344	47	0.61 (0.42–0.87)	0.65 (0.45–0.94)
Quartile 3 (>99.7–144.9)	344	33	0.45 (0.30–0.68)	0.52 (0.34–0.79)
Quartile 4 (>144.9)	344	35	0.51 (0.34–0.77)	0.53 (0.36–0.80)
p_trend_^c^			0.0004	0.0006
*MET-hours/week of sports activities* ^*d*^				
0	708	150	1.00 (Ref.)	1.00 (Ref.)
> 0–10	146	10	0.42 (0.22–0.81)	0.41 (0.22–0.80)
> 10–20	261	25	0.56 (0.37–0.86)	0.58 (0.37–0.89)
> 20	261	15	0.33 (0.19–0.56)	0.34 (0.20–0.59)
p_trend_^c^			<0.0001	<0.0001
*MET-hours/week of cycling activities* ^*d*^				
0	503	102	1.00 (Ref.)	1.00 (Ref.)
> 0–10	236	31	0.75 (0.50–1.14)	0.80 (0.52–1.22)
> 10–20	241	27	0.71 (0.45–1.10)	0.90 (0.57–1.41)
> 20	396	40	0.61 (0.42–0.90)	0.85 (0.57–1.27)
p_trend_^c^			0.02	0.52
*MET-hours/week of walking activities* ^*d*^				
0–10	409	75	1.00 (Ref.)	1.00 (Ref.)
> 10–20	386	56	0.82 (0.58–1.16)	0.83 (0.58–1.19)
> 20–30	297	37	0.65 (0.44–0.96)	0.67 (0.45–1.00)
> 30	284	32	0.62 (0.41–0.94)	0.65 (0.43–1.00)
p_trend_^c^			0.01	0.03
*MET-hours/week of gardening activities* ^*d*^				
0	297	69	1.00 (Ref.)	1.00 (Ref.)
> 0–10	358	48	0.72 (0.49–1.06)	0.81 (0.55–1.20)
> 10–20	264	23	0.38 (0.23–0.61)	0.41 (0.25–0.68)
> 20	457	60	0.55 (0.38–0.79)	0.62 (0.42–0.91)
p_trend_^c^			0.003	0.01
*MET-hours/week of housework, home repair, and stair climbing activities* ^*d*^				
0–10	177	45	1.00 (Ref.)	1.00 (Ref.)
> 10–20	221	29	0.60 (0.37–0.95)	0.65 (0.40–1.05)
> 20–30	194	29	0.69 (0.43–1.10)	0.72 (0.45–1.17)
> 30	784	97	0.70 (0.48–1.01)	0.83 (0.55–1.23)
p_trend_^c^			0.35	0.99
*Hours of sleeping at night* ^*e*^				
≤ 6	294	42	1.03 (0.72–1.45)	0.97 (0.68–1.38)
7–8	933	132	1.00 (Ref.)	1.00 (Ref.)
≥ 9	149	26	1.08 (0.71–1.65)	0.99 (0.65–1.53)
p_trend_^c^			0.95	0.87
*Hours of sleeping at day* ^*e*^				
0	607	57	1.00 (Ref.)	1.00 (Ref.)
> 0 – <1	98	7	0.58 (0.26–1.27)	0.53 (0.24–1.17)
1 – <2	558	94	1.19 (0.85–1.68)	1.17 (0.82–1.65)
≥ 2	113	42	2.63 (1.72–4.02)	2.22 (1.43–3.44)
p_trend_^c^			<0.0001	0.0004
*Hours/day of watching TV* ^*f*^				
≤ 2	480	55	1.00 (Ref.)	1.00 (Ref.)
> 2 – <4	414	59	1.16 (0.80–1.68)	1.23 (0.85–1.79)
≥ 4	482	86	1.28 (0.91–1.80)	1.45 (1.02–2.06)
p_trend_^c^			0.16	0.04

*Abbreviations*: *BMI* body mass index, *CRC* colorectal cancer, *MET* metabolic equivalent of task; *TV* television

^a^Estimated with Cox proportional hazards regression models

^b^Adjusted for sex, age at physical activity assessment, BMI, survival time from CRC diagnosis until physical activity assessment, tumor location, occurrence of metastases, occurrence of other cancer, chemotherapy, smoking status, alcohol intake, (time x age), (time x BMI), and (time x metastases)

^c^Calculated by modeling the median value of physical activities, sleeping time, or TV watching categories as a continuous variable

^d^multivariable-adjusted models mutually adjusted for ‘cycling’, ‘sports’, ‘walking’, ‘gardening’, and ‘housework, home repair, and stair climbing’

^e^multivariable-adjusted models mutually adjusted for hours of sleeping at night and hours of sleeping at day

^f^multivariable-adjusted models additionally adjusted for total physical activity

Considering individual types of physical activity, sports showed the strongest inverse association with all-cause mortality (HR: 0.34; 95% CI: 0.20–0.59, comparing >20 with 0 MET-hours/week, p_trend_ < 0.0001), independent of other types of physical activity. Similarly, also MET-hours of walking (HR: 0.65; 95% CI: 0.43–1.00 for >30 vs. 0–10 MET-hours/week, p_trend_ = 0.03) and of gardening activities (HR: 0.62; 95% CI: 0.42–0.91 for >20 vs. 0 MET-hours/week, p_trend_ = 0.01) were associated with survival in multivariable-adjusted models (Table 2). No statistically significant association with all-cause mortality after multivariable adjustment could be observed for cycling (p_trend_ = 0.52) and for the combination of activities from housework, home repair, and stair climbing (p_trend_ = 0.99; Table 2).

Notable differences with respect to their association with all-cause mortality were observed between hours of sleeping at night and hours of sleeping at day (Table 2). Whereas the sleep duration at night displayed no statistically significant association with survival time, individuals who slept ≥2 h during the day had more than twice the risk of dying (HR: 2.22; 95% CI: 1.43–3.44, p_trend_ = 0.0004) compared to individuals who did not sleep at day. Furthermore, ≥4 h/day spent watching TV displayed a significant 45% higher all-cause mortality compared with ≤2 h/day of TV viewing (HR: 1.45; 95% CI: 1.02–2.06, p_trend_ = 0.04; Table 2).

### Stratified analyses by potential effect modifiers

**Fig. 3:**
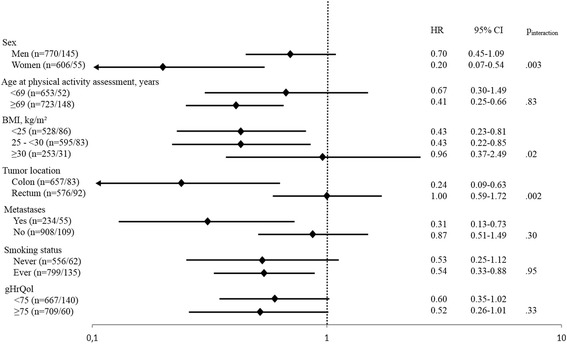
HRs and 95% CIs for all-cause mortality in 1376 CRC survivors comparing the fourth to the first quartile of total physical activity, stratified by potential effect modifiers; for each stratum the total number of individuals/number of deaths is shown; HRs and 95% CIs were estimated with Cox proportional hazards models, adjusted for sex, age at physical activity assessment, BMI, survival time from CRC diagnosis until physical activity assessment, tumor location, occurrence of metastases, occurrence of other cancer, chemotherapy, smoking status, alcohol intake, (time x age), (time x BMI) and (time x metastases), except the stratifying variable; p_interaction_ was calculated by entering into the model an interaction term of total physical activity as a continuous variable and the stratifying covariate; cutpoint for age at physical activity assessment and gHrQol was the respective median value. Abbreviations: BMI, body mass index; CRC, colorectal cancer; gHrQol, global health-related quality of life

The stratification by potential effect modifiers revealed significant quantitative interactions by sex, BMI, and tumor location (Fig. 3). The inverse association between total physical activity and all-cause mortality was stronger in women than in men (p_interaction_ = 0.003), stronger in individuals with a lower BMI (e.g. <25 kg/m^2^ or 25 - < 30 kg/m^2^) than in individuals with a higher BMI (e.g. ≥30 kg/m^2^) (p_interaction_ = 0.02), and stronger in individuals with a colon carcinoma than in individuals with a rectum carcinoma (p_interaction_ = 0.002). There was no evidence for a statistically significant interaction by age, occurrence of metastases, smoking status, and gHrQol, although the association was slightly stronger in older than in younger individuals and in individuals with metastases than in those without metastases (Fig. 3).

### Sensitivity analyses

After excluding participants who died within 12 months of physical activity assessment (*n* = 19), the results remained essentially unchanged (Additional file 1: Table S1). After exclusion of individuals who reported a diagnosis of metastases (*n* = 234), the association of physical activity with survival was a little weaker and slightly failed to reach statistical significance (probably because of the smaller sample size), but the inverse pattern of association was comparable to the overall sample (Additional file 1: Table S2). In another sensitivity analysis, we additionally adjusted the multivariable-adjusted Cox regression models and the restricted cubic spline regression for gHrQol. However, results did not change substantially. We observed that all associations were slightly attenuated and that the relation of walking with survival was rendered statistically nonsignificant (HR: 0.73; 95% CI: 0.47–1.14), upon adjustment for gHrQol. The restricted cubic spline regression still revealed a nonlinear trend (p_nonlinear_ = 0.05) (data not shown). Excluding participants who reported 0 MET-hours/week of total physical activity (*n* = 8) did not change the results appreciably (data not shown). Additionally adjusting the association of TV viewing with all-cause mortality for total energy intake did not cause any change in the results (data not shown).

## Discussion

### Principal observations

In this cohort of 1376 long-term CRC survivors, higher postdiagnostic total physical activity was associated with lower all-cause mortality. The observed association emerged as nonlinear with an approximately similar reduction of all-cause mortality for individuals with moderate and for individuals with high physical activity as compared to individuals with lower levels of activity. We identified significant effect modification by sex, BMI, and tumor location in the sense that the observed association between total physical activity and all-cause mortality was stronger in women, in individuals with a lower BMI, and in individuals with a colon carcinoma. Regarding individual types of physical activity, sports, walking, and gardening were particularly strongly inversely related to all-cause mortality. A greater amount of sleeping during the day was associated with shorter survival, whereas the amount of sleep at night was not associated with survival. More hours per day spent watching TV were associated with a higher all-cause mortality in our CRC survivor cohort.

### In the context of the current literature

Our observation of a significant inverse association of postdiagnostic physical activity with all-cause mortality is consistent with a recent meta-analysis of 7 prospective cohort studies of patients with CRC, reporting a summary RR of 0.71 (95% CI: 0.63–0.81) for total mortality, associated with high levels versus low levels of physical activity [37]. With respect to the results obtained in individual cohorts, a 42% (95% CI: 0.47–0.71) reduction in the relative risk for all-cause mortality associated with 8.75 or more MET-hours/week (compared to less than 3.5 MET-hours/week) of recreational physical activity was reported in 2293 CRC survivors [17]. Of note, the time intervals between CRC diagnosis and physical activity assessment were much shorter in most prior studies (range: 5 months to 4.2 years median) [16–23] as compared to our study (6 years median). Thus, we expand the existing evidence by showing that the relation between higher physical activity and better overall survival is also present in long-term survivors of CRC.

Furthermore, to our knowledge, our study is the first one to investigate the association of different types of postdiagnostic physical activity (e.g. walking, cycling, sports, gardening, and housework) with mortality of CRC survivors. However, a randomized controlled trial investigated different intensities of physical activity with cardiorespiratory fitness and body composition in CRC survivors and observed a significantly enhanced cardiorespiratory fitness, increased lean mass, and decreased fat mass in individuals with high- vs. moderate-intensity exercise [38].

With respect to the association of watching TV with all-cause mortality, a prior study (*n* = 1759 participants) reported likewise an increased risk for all-cause mortality in individuals with ≥4 h per day of TV viewing compared to individuals with 0–2 h of TV watching per day (HR: 1.25; 95% CI: 0.93–1.67) [16]. Similarly, an HR of 1.45 (95% CI: 0.73–2.87) for ≥21 h/week of watching TV compared to 0–6 h of TV viewing was reported in a sample including 714 male CRC survivors [24]. However, in these two studies, statistical significance could not be reached.

In our analyses, the effect of total physical activity on all-cause mortality differed by sex, BMI, and tumor location. Specifically, the association was stronger in women, which is in line with observations in a study of 879 CRC survivors in Western Australia [20]. Furthermore, individuals with a lower BMI displayed a stronger association of physical activity with overall survival as compared to individuals with a higher BMI. Concerning this interaction, other studies revealed heterogeneous results [18–20]. In our cohort, individuals with a colon tumor had a stronger association of physical activity with overall survival than individuals with a rectum tumor. A similar but nonsignificant tendency was reported in an Australian study [19]. Additionally, in the European Prospective Investigation into Cancer and Nutrition, physical activity was associated with a reduction of colon cancer incidence, but not of rectum cancer incidence [32].

The average level of physical activity, measured in MET-hours per week, in our sample was higher than in most of the other studies of CRC survivors [17, 21, 23]. It has to be kept in mind, though, that in our cohort nearly all activities (leisure time activities (sports, cycling, walking), gardening, and housework activities (housework, home repair, stair climbing)) were enquired and included in the analyses, whereas most prior studies relied only on leisure time activities. Additionally, regarding the median age of 69 years, it can be assumed that the vast majority of our participants were no longer engaged in occupational activities when physical activity was assessed, so that almost every kind of usual activity should be recorded when leisure time physical activity and housework/gardening activities are gathered.

### Potential explanations for the observed associations

Several beneficial health effects of physical activity have been reported, including improvements in metabolism, inflammatory processes, and vascular and cardiac function. Specifically, greater insulin sensitivity and lower levels of insulin [39] were related to increased physical activity. In prospective studies, higher circulating insulin and C-peptide levels have been associated with CRC risk [40], angiogenesis, tumor growth, and anti-apoptosis [41]. Another potential mechanism is that physical activity decreases inflammatory adipocytokines and increases circulating concentrations of anti-inflammatory cytokines, which could affect cancer incidence and mortality [42]. Physical activity also improves structure and function of the cardiovascular system, e.g., by lowering blood pressure [7] and by positively affecting vascular remodeling [43]. In this context, a small intervention study in 47 CRC survivors revealed that a 4-week exercise program of high intensity as compared to moderate intensity led to a significant improvement in cardiorespiratory fitness and body composition [38]. The differences in the association between the different types of physical activity with all-cause mortality cannot be fully explained with our dataset because we do not know the exact type and intensity of activity within a given activity group (e.g. in sports, gardening, housework). As outlined in the methods section, we obtained the duration of each activity and then multiplied it with a recommended averaged MET-value [31, 32]. One potential explanation for the observed differences between the different types of activity could be that sports activities conducted by the participants included more high-intensity exercise as compared to cycling activities and that gardening activities may include more high-intensity exercise as compared to household activities. But these premises require further investigations with more detailed information on intensity level and type of activity. Another beneficial effect of gardening (as compared to household activities) could also be the outdoor exercise in fresh air with more sunlight exposure leading to an increased vitamin D synthesis. Previous studies reported an association between higher plasma vitamin D levels and lower all-cause mortality in CRC survivors [44, 45]. A high level of walking activities might reflect an active lifestyle in general which may have led to the reduction in all-cause mortality with more MET-hours/week of walking in our cohort.

With respect to the observed association between TV viewing and all-cause mortality, higher amounts of time spent watching TV have been associated with higher levels of cardiometabolic biomarkers and increased risk of cardiovascular disease and obesity [46], diabetes [47], and all-cause mortality [48]. One of the potential mechanisms for the observed association includes greater amounts of sedentary time in individuals watching more TV and a higher consumption of energy-dense food [36]. However, in a sensitivity analysis, we additionally adjusted the association of TV viewing with all-cause mortality for total energy intake and observed no differences in HRs and 95% CIs.

The observed association between more hours of sleeping at day and higher all-cause mortality could be explained by reduced physical activity and higher sedentary time leading to adverse biological consequences as mentioned above. However, it is also plausible that reverse causality may have influenced this association. It cannot be ruled out, that individuals with a worse health status spend more time sleeping at day due to uncomfortable feeling and lack of energy.

Reverse causality might also play a role for the association between physical activity and mortality in general (e.g. less physical activity due to indisposition). Although we performed several sensitivity analyses to address this point (additional adjustment for gHrQol; exclusion of participants who died within 12 months after physical activity assessment; exclusion of individuals with 0 MET-hours/week of total physical activity), and the results remained largely unchanged in these analyses, reverse causality cannot entirely be ruled out.

The nonlinearity of the association between total physical activity and all-cause mortality reveals that compared to nearly no activity, a moderate level is associated with a lower risk of all-cause mortality whereas the differences in mortality risk between high activity and moderate activity were not so prominent. Thus, physical activity at all compared to nearly none might be beneficial for CRC survivors with moderate and high levels of physical activity conferring approximately similar benefits with respect to survival.

The difference in the association of physical activity with all-cause mortality between men and women and between individuals with a lower BMI and those with a higher BMI might be due to a generally healthier lifestyle in women than in men [49] and in individuals with a lower BMI, e.g., in the normal range or in the overweight category, as compared to individuals with a BMI in the obese category [50]. Additionally, obese individuals might be more prone to misreport physical activity which may have led to the lack of association in the obese participant group [51]. Regarding the difference between colon and rectum carcinoma, a hypothesized mechanism is that physical activity might accelerate bowel motility more intensely in the colon than in the rectum which can affect the gastrointestinal transit time and the time in which potential carcinogens have contact with the mucosa [52].

### Strengths and limitations

Strengths of our study include the prospective design with a long follow-up period, a relatively large sample, and a comprehensive ascertainment of physical activity, its subtypes, and vital status.

However, some limitations need to be considered. We only had information available on all-cause mortality, but not on disease-specific mortality. Therefore, future studies on the association of physical activity, especially of different types of activities, with CRC-specific mortality are warranted. The CRC diagnosis of our study participants occurred at a median of 6 years prior to physical activity assessment, which is why we characterize them as long-term cancer survivors. Thus, the generalizability of our observations to all CRC patients is unknown. Additionally, information on tumor stage and comorbidities were not available in our cohort. We only had information on metastases and other cancers. Though, a recent study that investigated the association between prediagnostic physical activity and survival did not find any differences in the results after adjusting for comorbidities in a sensitivity analysis [33]. We also were not able to adjust the association between sleep during daytime and survival for medication use, even though some medication could influence fatigue and sleeping time as well as mortality. Furthermore, we had no information on prediagnostic physical activity. However, a previous study reported a significant association of postdiagnostic physical activity with mortality independent of prediagnostic activities [16]. Moreover, reported activities, especially in the category of sports, are likely to vary between participants in type or intensity, which has not been assessed specifically. The data on clinical and lifestyle factors were self-reported which may have led to some information or recall bias. Nevertheless, a validation of self-reported clinical data against medical records in a subset of 181 patients revealed a concordance of about 87%.

## Conclusions

Our results strengthen the evidence on the association of higher postdiagnostic physical activity with reduced mortality risk in CRC survivors. Certain activity types might be primarily responsible for this association. The association of lifestyle factors (such as physical activity and sedentary behavior) after CRC diagnosis with survival is particularly interesting, because CRC survivors might be able to alter their behavior and actively improve their health outcome, a premise that could be addressed in further (interventional) studies. The fact that reverse causality is a common problem in observational studies underscores the need for randomized controlled trials of physical activity interventions in CRC survivors.

Furthermore, physical activity could be an attractive strategy to prevent cancer recurrence and to prolong life in cancer survivors because it potentially also prevents many other diseases which accumulatively appear in the elderly [53]. Based on the available evidence, it is reasonable to recommend CRC survivors to engage in regular physical activity.

## Additional file

Additional file 1: Table S1. Sensitivity Analysis (*n* = 1357): HRs and 95% CIs of all-cause mortality according to quartiles of physical activity after excluding individuals who died within 12 months after physical activity assessment (*n* = 19); **Table S2.** Sensitivity Analysis (*n* = 1142): HRs and 95% CIs of all-cause mortality according to quartiles of physical activity after excluding individuals with known occurrence of metastases (*n* = 234). (DOCX 20 kb)

## Abbreviations

BMI: Body mass index
CRC: Colorectal cancer
FFQ: Food frequency questionnaire
gHrQol: Global health-related quality of life
HrQol: Health-related quality of life
MET: Metabolic equivalent of task
TV: Television
