# Supplementary material for: Postdiagnostic physical activity, sleep duration, and TV watching and all-cause mortality among long-term colorectal cancer survivors: a prospective cohort study
Source: BMC Cancer. 2017 Oct 25;17:701. doi: 10.1186/s12885-017-3697-3 (PMC5657114; doi:10.1186/s12885-017-3697-3)
Supplement: Additional file 1: Table S1. — Sensitivity Analysis (n = 1357): HRs and 95% CIs of all-cause mortality according to quartiles of physical activity after excluding individuals who died within 12 months after physical activity assessment (n = 19); Table S2. Sensitivity Analysis (n = 1142): HRs and 95% CIs of all-cause mortality according to quartiles of physical activity after excluding individuals with known occurrence of metastases (n = 234). (DOCX 20 kb) [file 12885_2017_3697_MOESM1_ESM.docx]

Additional file 1

**Postdiagnostic physical activity, sleep duration, and TV watching
and all-cause mortality among long-term colorectal cancer survivors: a prospective cohort study**

**Authors:** Ilka Ratjen, Clemens Schafmayer, Romina di Giuseppe, Sabina Waniek, Sandra Plachta-Danielzik, Manja Koch, Greta Burmeister, Ute Nöthlings, Jochen Hampe, Sabrina Schlesinger, Wolfgang Lieb

**Address for correspondence:** Wolfgang Lieb, MD MSc, Institute of Epidemiology, Christian-Albrechts-University of Kiel, University Hospital Schleswig-Holstein, Niemannsweg 11 (Haus 1), 24105 Kiel (Germany), Tel.: +49 431 500-30200, Fax: +49 431 500-30204, Email: wolfgang.lieb@epi.uni-kiel.de.

**Table S1** Sensitivity Analysis (n=1357): HRs^1^ and 95% CIs of all-cause mortality according to quartiles of physical activity after excluding individuals who died within 12 months after physical activity assessment (n=19)

|  | **Total no. of individuals** | **No. of deaths** | **Age- & sex-adjusted HR (95% CI)** | **Multivariable-adjusted^2^ HR (95% CI)** |
| --- | --- | --- | --- | --- |
| ***MET-hours/week of total physical activity*** |  |  |  |  |
| Quartile 1 (0-65.2) | 339 | 74 | 1.00 (Ref.) | 1.00 (Ref.) |
| Quartile 2 (>65.2-100.7) | 339 | 42 | 0.65 (0.44-0.95) | 0.67 (0.46-0.99) |
| Quartile 3 (>100.7-147.0) | 340 | 32 | 0.52 (0.34-0.79) | 0.58 (0.38-0.89) |
| Quartile 4 (>147.0) | 339 | 33 | 0.58 (0.38-0.88) | 0.59 (0.39-0.90) |
| p_trend_^3^ |  |  | 0.006 | 0.008 |

^1^ Estimated with Cox proportional hazards models.

^2^ Adjusted for sex, age at physical activity assessment, BMI, survival time from CRC diagnosis until physical activity assessment, tumor location, occurrence of metastases, occurrence of other cancer, chemotherapy, smoking status, alcohol intake, (time x age), (time x BMI), and (time x metastases).

^3^ Calculated by modeling the median value of total physical activity quartiles as a continuous variable.

Abbreviations: BMI, body mass index; CRC, colorectal cancer; Ref., reference.

**Table S2** Sensitivity Analysis (n=1142): HRs^1^ and 95% CIs of all-cause mortality according to quartiles of physical activity after excluding individuals with known occurrence of metastases (n=234)

|  | **Total no. of individuals** | **No. of deaths** | **Age- & sex-adjusted HR (95% CI)** | **Multivariable-adjusted^2^ HR (95% CI)** |
| --- | --- | --- | --- | --- |
| ***MET-hours/week of total physical activity*** |  |  |  |  |
| Quartile 1 (0-65.5) | 285 | 58 | 1.00 (Ref.) | 1.00 (Ref.) |
| Quartile 2 (>65.5-100.2) | 286 | 35 | 0.71 (0.46-1.08) | 0.72 (0.47-1.11) |
| Quartile 3 (>100.2-143.5) | 286 | 25 | 0.53 (0.33-0.85) | 0.60 (0.37-0.97) |
| Quartile 4 (>143.5) | 285 | 27 | 0.66 (0.41-1.05) | 0.65 (0.40-1.04) |
| p_trend_^3^ |  |  | 0.04 | 0.05 |

^1^ Estimated with Cox proportional hazards models.

^2^ Adjusted for sex, age at physical activity assessment, BMI, survival time from CRC diagnosis until physical activity assessment, tumor location, occurrence of other cancer, chemotherapy, smoking status, alcohol intake, and (time x age).

^3^ Calculated by modeling the median value of total physical activity quartiles as a continuous variable.

Abbreviations: BMI, body mass index; CRC, colorectal cancer; Ref., reference.
